# Markers of bone turnover after 12 months of exercise in patients with chronic kidney disease 3–5: a post-hoc analysis of RENEXC – a randomized controlled trial

**DOI:** 10.1186/s12882-025-04501-9

**Published:** 2025-10-13

**Authors:** Vaida Petrauskiene, Matthias Hellberg, Philippa Svensson, Naomi Clyne

**Affiliations:** Lund University, Skåne University Hospital, Faculty of Medicine, Department of Clinical Sciences Lund, Nephrology , Lund, Sweden

**Keywords:** Bone markers, Chronic kidney disease, Exercise, Procollagen type I N-terminal propeptide, Tartrate-resistant acid phosphatase isoform 5b

## Abstract

**Background:**

Bone and mineral disorders are common in patients with chronic kidney disease (CKD), leading to poor quality of life, high fracture risk, and increased morbidity and cardiovascular mortality. Parathyroid hormone (PTH) and bone-specific alkaline phosphatase (bALP) are frequently used to assess bone turnover, but markers such as procollagen type I N-terminal propeptide (intact PINP) and tartrate-resistant acid phosphatase isoform 5b (TRAP5b), which reflect bone formation and resorption, may provide more specific insights into bone remodeling. This study aims to investigate the effects of balance and strength exercises on bone turnover markers in patients with CKD not undergoing kidney replacement therapy.

**Methods:**

This study is a post-hoc sub-analysis of the RENEXC trial, a randomized controlled exercise intervention lasting 12 months. A total of 151 CKD stage 3–5 patients were randomly assigned to either strength or balance exercise, both combined with endurance exercise. Exercise intensity was monitored using the Borg Rating of Perceived Exertion scale. Bone turnover markers, including intact PINP and TRAP5b, were measured at baseline and after 12 months.

**Results:**

Over 12 months, within-group changes in bone turnover markers were not statistically significant. In the strength group, intact PINP showed a non-significant increase and TRAP5b a slight decrease, while in the balance group intact PINP tended to decrease and TRAP5b remained stable. After adjustment for baseline values, between-group differences at 12 months were small and non-significant for both markers. Overall adjusted means were 78.7 µg/L for intact PINP and 1.87 U/L for TRAP5b, with no significant effects of treatment group, sex, or CKD stage. Additionally, both exercise groups showed an increase in the proportion of patients with TRAP5b values indicative of low bone turnover, suggesting a possible protective effect on bone resorption.

**Conclusions:**

Neither strength nor balance exercise significantly altered bone turnover markers over 12 months in patients with CKD. Nonetheless, both interventions were associated with more participants exhibiting TRAP5b values consistent with low bone turnover, indicating a potential role in mitigating bone resorption. These findings warrant further investigation into the long-term effects of exercise on bone health in CKD, with a particular focus on the mechanisms underlying these responses.

**Trial registration:**

NCT02041156 at http://www.ClinicalTrials.gov. Date of registration January 20, 2014. Retrospectively registered.

## Background

Bone and mineral disorders are common complications in patients with chronic kidney disease (CKD) resulting in poor quality of life, high fracture risk, increased morbidity and cardiovascular mortality. Parathyroid hormone (PTH) has long been the preferred surrogate biomarker of bone turnover together with bone specific alkaline phosphatase (bALP) and is recommended for routine clinical use in the most recent Kidney Disease-Improving Global Outcomes (KDIGO) guidelines [1]. Bone turnover markers, released from bone tissue—such as procollagen fragments or enzymes secreted by bone cells—could be superior to PTH, a regulator of bone metabolism, in enhancing our understanding of bone remodelling processes [2]. They show acceptable diagnostic accuracy for bone turnover and in everyday clinical practice could be seen as an accessible alternative to bone biopsy, which is still considered to be the gold standard, in diagnosing renal osteodystrophy [2].

In CKD, certain bone turnover markers may accumulate due to reduced renal clearance, limiting their usefulness; therefore, selecting suitable markers requires caution. Procollagen type I N-terminal propeptide (PINP) is a reference marker of bone formation as it is released as collagen is deposited in the bone matrix [3]. Monomeric fragments accumulate in CKD, necessitating the use of specific assays for the intact, trimeric form of PINP (intact PINP) in this specific group of patients [4].

Tartrate-resistant acid phosphatase isoform 5b (TRAP5b) is released from osteoclasts and is considered a highly specific marker of bone resorption that is not affected by changes in renal function [5].

Very few studies, mostly with small sample sizes and examining patients on dialysis, studied the relationship of exercise and changes in bone specific biomarkers and did not show significant associations [6, 7]. Although positive effects of resistance exercise on the regulation of bone formation and resorption biomarkers in healthy subjects has been described [8, 9], there are to our knowledge no studies which have examined the influence of exercise on changes in bone specific biomarkers in patients with CKD not on kidney replacement therapy (KRT).

In a previous sub-study of RENEXC, a randomised controlled head-to-head trial examining two different exercise regimes, balance or strength both in combination with endurance exercise, during 12 months of intervention, we explored the possible effects of exercise on bone mineral density in patients with CKD stages 3 to 5 not on KRT. We showed that balance exercise seemed to be superior in maintaining and improving whole body bone mineral density compared with strength exercise [10]. We concluded that balance exercise in combination with endurance exercise appeared to influence bone metabolism processes positively in these patients.

The aim of this study was to investigate the effects of balance- and strength exercise (both in combination with endurance exercise) on markers of bone metabolism in patients with CKD stages 3–5 not on KRT.

## Materials and methods

### Study design

This study is a post-hoc analysis of the RENEXC trial, a prospective randomized controlled exercise trial, with an intervention period of 12 months. There were no changes to protocol after the trial began. Complete study design and primary analysis of RENEXC data have been reported previously [11]. Some information on study design and methods is repeated here for clarity.

All incident and prevalent patients at the Department of Nephrology in Lund, Skåne University Hospital with an estimated GFR < 30 ml/min/1.73 m², age ≥ 18 years, all kidney diseases and any number of comorbidities were eligible for participation in the study. Orthopaedic impediment, severe neurological dysfunction, inability to understand patient information and instructions, estimated start of KRT within 12 months of study start were exclusion criteria.

The study was approved by the Regional Ethical Review Board in Lund (registration number 2011/369) and adhered to the Helsinki Declaration. All participants gave informed consent prior to inclusion after having received written and oral information.

### Intervention

151 patients were randomised to either strength or balance exercise both together with endurance exercise. The exercises were individually prescribed, monitored by a physiotherapist, and self-administered at home or at a nearby gym. The Borg Rating of Perceived Exertion scale (RPE) was used to prescribe exercise intensity and to monitor progress [12]. The prescribed weekly exercise duration was 150 min, comprising 60 min per week of endurance exercise at a RPE of 13–15 for both groups combined with 90 min per week of either strength or balance exercise at a RPE of 13–17 per exercise set.

All patients were tested at baseline (T0) and after 12 months (T12).

Measures of physical performance were the primary outcomes.

### Physical performance

Overall endurance was tested with the 6-minute walk test (6-MWT) [13]. Muscular endurance and fatigability in the proximal leg muscles were tested with the 30-seconds sit-to-stand test (30-STS) [14]. Balance was tested with functional reach (FR) [15, 16]. Neuromuscular function and strength in the lower extremities were tested with isometric quadriceps strength (ISQ) [15, 16].

### Comorbidity score

Comorbidity was assessed with the Davies Comorbidity Score, which is a clinical tool used to predict mortality risk by assessing the presence and severity of coexisting medical conditions [17], at baseline.

### Laboratory analyses

Plasma 25 hydroxyvitamin D (25(OH)D) was analysed using liquid chromatography-mass spectrometry. Glomerular filtration rate (GFR) was measured using iohexol clearance. Calcium, phosphate were measured using standard laboratory techniques and performed at the Department of Clinical Chemistry, Laboratory Medicine Skåne, which is accredited by SWEDAC (ISO 15189:2012). ALP was measured using a colorimetric method on Cobas instruments (Roche Diagnostics, Basel, Switzerland) with a reference range of 0.6–1.8 µkat/L. PTH was measured using an electrochemiluminescence immunoassay (ECLIA) method on a Cobas instrument (Roche, Mannheim, Germany) with a reference range of 1.6–6.9 pmol/L.

Average values of calcium, phosphate, PTH, and ALP were calculated from measurements at baseline and months 4, 8, and 12, while 25(OH)D averages were calculated using only baseline and end-of-study measurements. These averages were classified as within, above, or below the respective reference intervals for analysis.

Trimeric procollagen- type I N-propeptide (intact PINP, assay range 2.0–4600 µg/L) and tartrate-resistant acid phosphatase isoform 5b (TRAP5b, assay range 0.9–56 U/L) were measured with ImmunoDiagnostic System iSYS instrument (IDS, Boldon, UK). The plasma was collected fasting and stored at − 80 °C. Values of intact PINP above upper limit of quantification of the assay were determined after dilution. For intact PINP the intra and inter assay variations are both at maximum 8%. For TRAcp-5b the intra and inter assay variations are both at maximum 12%.

### Evaluation of bone turnover markers

To define between group changes after the study period Δ intact PINP and Δ TRAP5b values (T12 value – T0 value) were calculated. For 10 patients, complete data were not available, as one of the two samples required for paired analysis was missing; these cases were therefore excluded from the analysis. For more detailed analyses after the study period, we used calculated cutoffs for high and low turnover reported by Jørgensen et al. [2]. Thresholds of intact PINP > 120.7 µg/L and TRAP5b > 5.05 U/L have been suggested as indicative of high bone turnover. Conversely, values of intact PINP < 49.8 µg/L and TRAP5b < 3.44 U/L are consistent with low turnover and due to their high negative predictive value, may be particularly informative for excluding high turnover states.

Due to limited data on TRAP5b and intact PINP thresholds in CKD G3–5, patients were also classified as above or below the pre-menopausal median values of 3.1 U/L for TRAP5b and 36 µg/L for intact PINP [18, 19].

### Randomisation

Patients were randomized using ProcPlan in SAS, SAS Institute Cary NC. The statistician generated the random allocation sequence, the nephrologist enrolled patients and the physiotherapist assigned participants to intervention. Patients were included and allocated sequential treatment according to a list that only the research physiotherapist had access to. Both interventions comprised endurance exercise. The difference between the treatment arms was that one group was allocated strength exercise and one group balance exercise.

### Statistical analyses

Statistical analyses were performed using the SPSS for Windows software program version 24.0. Per-protocol analysis was employed including only patients who completed the treatment originally allocated. Variables were expressed as frequencies, percentages for discrete factors. Continuous factors are presented as means ± standard deviations or medians [minimum and maximum]. The two-tailed chi-square and Wilcoxon tests was used for categorical variables. The two-tailed Student’s t-test or Mann-Whitney test for continuous variables, according to data distribution. The paired sample T-test was used for parametric variables. ANCOVA was used to compare groups adjusting for baseline values, and a general linear model was applied to assess the effects of treatment group, sex, and GFR stage on outcomes while controlling for baseline measurements. For all comparisons a p value < 0.05 was considered significant.

## Results

**Fig. 1:**
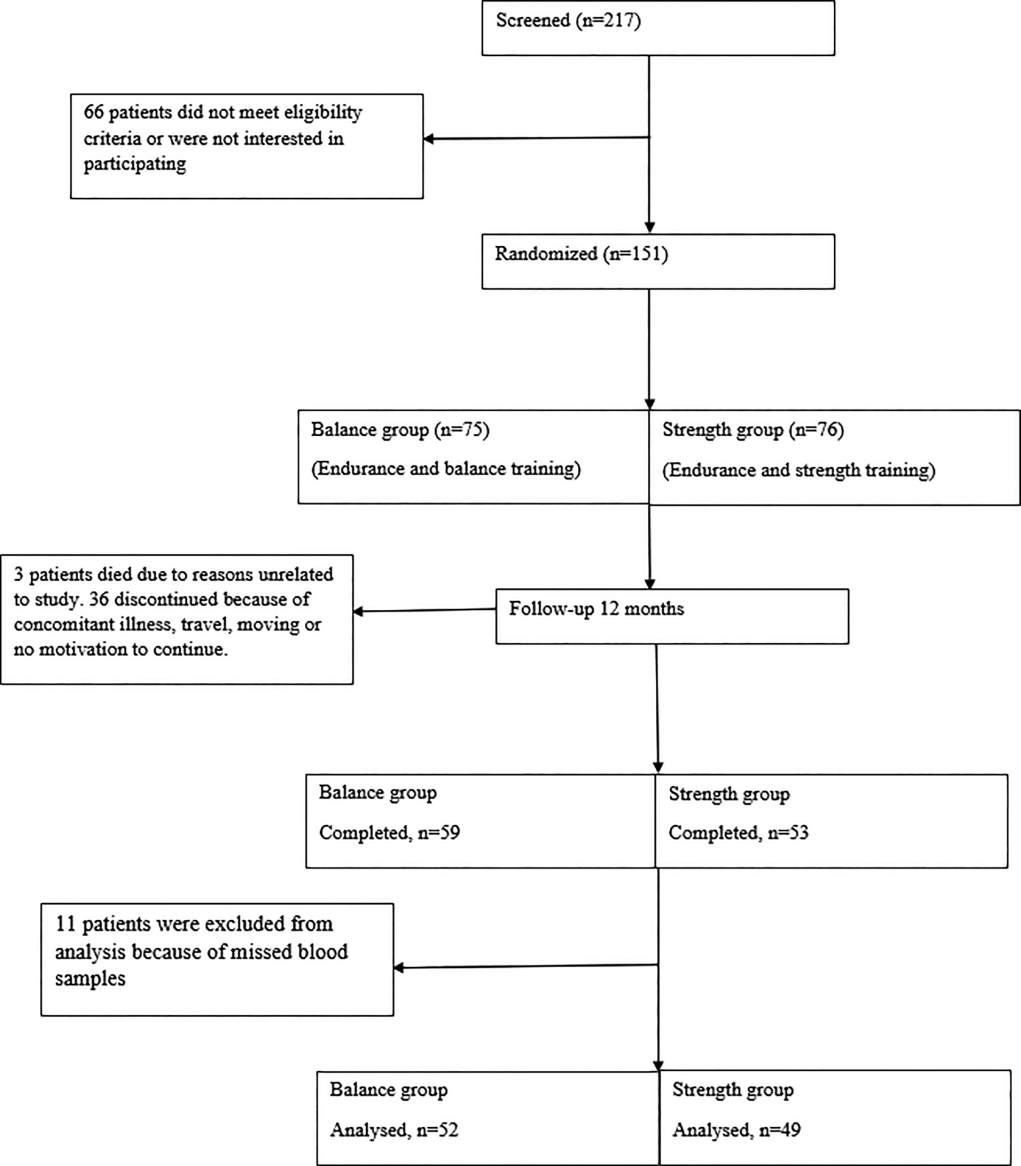
CONSORT diagram

A total of 217 patients were screened and 151 patients were randomized. 112 patients completed the whole exercise period of whom 11 patients were excluded from this study because of missing blood samples at both points. Consequently, results from 101 (73 men, 28 women) patients (mean age 68 ± 12.8 years) were analysed. Most of the patients were in CKD stage 4 (*n* = 67, 66%), 17 patients (17%) were in CDK 3 and 17 (17%) in CKD stage 5. 49 patients (37 men, 12 women) participated in the strength group, and 52 (36 men, 16 women,) in the balance group. The Consolidated Standards of Reporting Trials (CONSORT) flow diagram is presented in Fig. 1. Some clinical characteristics and laboratory data are presented in Table 1.

**Table 1:** Some clinical characteristics at baseline

	Units	Strength group *N* = 49	Balance group *N* = 52
Age	years	71 [23–84]	70 [27–87]
Male	n (%)	37 (76)	36 (69)
Weight	kg	83.7 ± 18.0	79.6 ± 16.7
Height	m	1.7 ± 0.1	1.7 ± 0.1
BMI	kg/m²	27.9 ± 5.2	27.0 ± 4.9
Diuretics*		35 (71)	36 (70)
PPI		14 (29)	13 (25)
Cinacalcet		1 (2)	0 (0)
Active vit D		28 (57)	34 (65)
Native vit D		3 (6)	6 (12)
Calcium based phosphate binders			
• Calcium carbonate		11 (23)	19 (37)
• Calcium acetate		1 (2)	1 (2)
Non-calcium-based phosphate binders			
• Sevelamer		6 (12)	1 (2)
• Lanthanum		1 (2)	3 (6)
Malignancy		4(8)	7 (14)
Ischemic heart disease		8 (16)	13 (25)
Peripheral vascular disease		11 (22)	9 (17)
Left ventricular dysfunction		4 (8)	6 (12)
Diabetes mellitus		15 (31)	10 (19)
Systemic collagen vascular disease		3 (6)	5 (10)
Other (e.g. hypertension)		33 (67)	38 (73)

Data are presented as mean ± SD or median [minimum- maximum]. BMI- body mass index, PPI- proton pump inhibitors, vit D- vitamin D. * 9 patients received hydrochlorothiazide, all others received loop diuretics

### Physical performance

The effects of 12 months of exercise on physical performance have been presented in detail previously and shall not be repeated here. Both groups improved overall endurance (6MWT), muscular endurance (30-STS), strength (IQS), and balance (FR) significantly, with no between-group differences [10, 11].

### Bone turnover markers

#### Overall changes

**Fig. 2:**
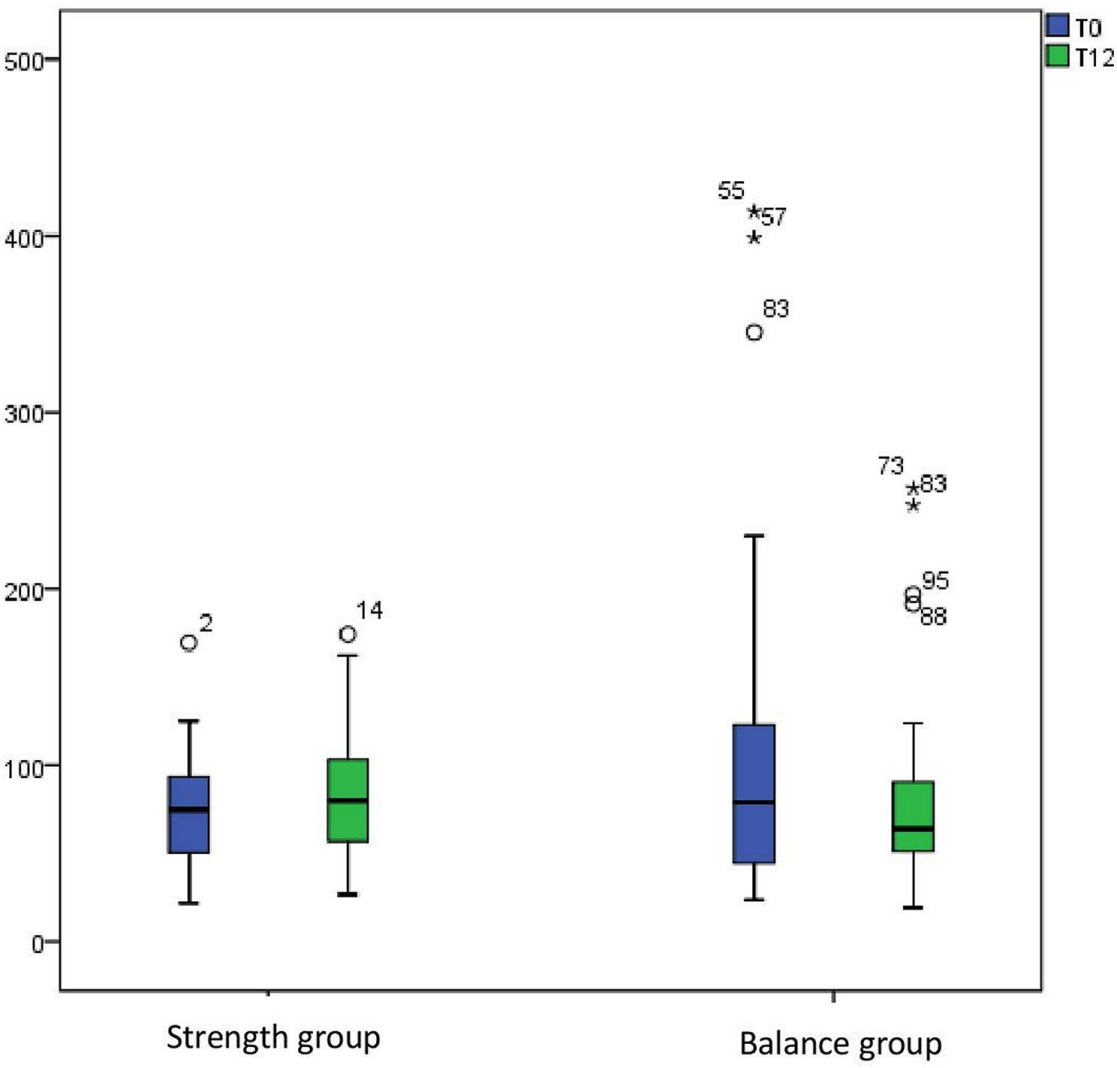
Distribution of intact PINP levels in the strength and balance groups at baseline (T0) and after 12 months (T12)

**Fig. 3:**
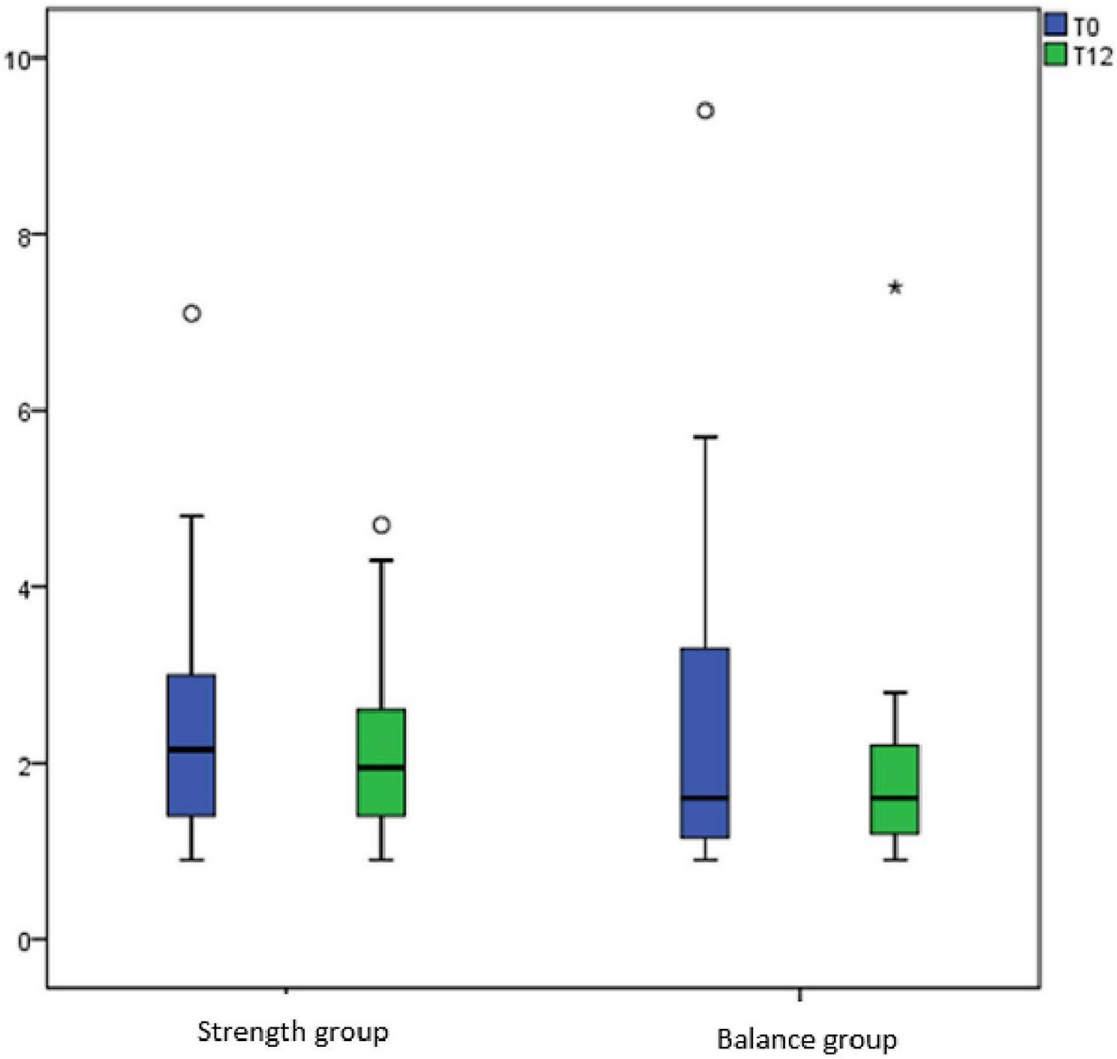
Distribution of TRAP5b levels in the strength and balance groups at baseline (T0) and after 12 months (T12)

Within-group changes over the 12-month study period were non-significant. In the strength group, intact PINP increased from 71.8 (21.8–169.5) to 79.7 (26.8–174.2; *p* = 0.20) and TRAP5b decreased from 2.2 (0.9–7.1) to 1.9 (0.9–4.7; *p* = 0.26). In the balance group, intact PINP decreased from 80.8 (23.8–413.6) to 63.8 (19.1–256.9; *p* = 0.15), while TRAP5b remained stable (1.6 [0.9–9.4] to 1.7 [0.9–7.4]; *p* = 0.16). Single values’ distribution at the start and after 12 months of the study within groups are presented in Figs. 2 and 3.

After adjusting for baseline values (ANCOVA), mean TRAP5b at 12 months was 2.07 (95% CI: 1.77–2.37) in the strength group and 1.81 (95% CI: 1.52–2.09) in the balance group, with an adjusted between-group difference of 0.27 (95% CI: −0.15–0.68; *p* = 0.21). Mean intact PINP at 12 months was 84.97 (95% CI: 71.03–98.91) in the strength group and 75.62 (95% CI: 62.60–88.64) in the balance group, yielding an adjusted between-group difference of 9.35 (95% CI: −9.86 − 28.55; *p* = 0.33).

### Stratification by CKD stage and sex

To explore potential differences in response to exercise based on kidney function, participants were stratified according to CKD stage. Patients with CKD stage 3 in the strength group demonstrated a significant decrease in TRAP5b over the 12-month study period (Table 2). No significant changes were observed in patients with CKD stages 4 or 5 in either exercise group.

**Table 2:** Bone markers at baseline (T0) and after 12 months of exercise (T12) stratified for CKD stages

	Strength group			Balance group			Whole group		
	T0	T12	*P*	T0	T12	*P*	T0	T12	*P*
Intact PINP (pg/mL)									
CKD 3	51 [22–170]	83 [40–151]	0.18	61 [39–156]	90 [60–124]	0.90	55 [22–170]	84 [40–150]	0.40
CKD 4	76 [32–120]	75 [27–174]	0.56	84 [26–414]	64 [19–257]	0.25	76 [22–414]	70 [19–257]	0.84
CKD 5	74 [33–114]	80 [38–160]	0.89	52 [23–345]	56 [25–247]	0.67	80 [24–345]	72 [25–247]	0.79
TRAP5b (U/L)									
CKD 3	2.1 [0.9–7.1]	1.6 [0.9–3.0]	**0.04**	3.4 [1.3–9.4]	2.4 [0.9–7.4]	0.50	2.9 [0.9–7.1]	2.0 [0.9–7.4]	0.89
CKD 4	2.2 [0.9–6.0]	2.2 [1.0–4.1]	0.53	1.6 [0.9–5.7]	1.7 [0.9–2.1]	0.40	1.8 [0.9–9.4]	1.8 [0.9–4.3]	0.16
CKD 5	2.4 [0.9–4.7]	1.1 [0.9–4.7]	0.18	1.3 [0.9–4.1]	1.5 [0.9–2.7]	0.67	2.0 [0.9–5.7]	1.5 [0.9–4.7]	0.18

Data are presented as median [minimum- maximum]. Intact PINP- N-terminal propeptide of type I procollagen, intact, TRAP5b - tartrate-resistant acid phosphatase 5b, CKD- chronic kidney disease

When examining sex-specific responses, women in the balance group showed a significant reduction in TRAP5b levels after 12 months of exercise (Table 3). No other sex-specific differences were observed for either bone turnover marker in the strength group or in men across groups.

**Table 3:** Bone markers at baseline (T0) and after 12 months of exercise (T12) stratified for sex

	Strength group			Balance group			Whole group		
	T0	T12	*P*	T0	T12	*P*	T0	T12	*P*
Intact PINP (pg/mL)									
Female	75 [22–169]	70 [31–174]	0.79	84 [24–414]	64 [19–124]	0.06	83 [22–414]	65 [1.9–174]	0.09
Male	70 [22–125]	82 [27–162]	0.10	69 [26–399]	64 [22–257]	0.58	70 [22–399]	74 [22–257]	0.47
TRAP5b (U/L)									
Female	1.9 [0.9–7.1]	1.6 [1–4.1]	0.45	2.2 [1–9.4]	1.7 [0.9–2.8]	**0.03**	2.1 [0.9–9.4]	1.6 [0.9–4.1]	**0.03**
Male	2.3 [0.9–6.0]	2.0 [0.9–4.7]	0.33	1.5 [0.9–5.7]	1.7 [0.9–7.4]	0.90	1.8 [0.9–6.0]	1.8 [0.9–7.4]	0.59

Data are presented as median [minimum- maximum]. Intact PINP- N-terminal propeptide of type I procollagen, intact, TRAP5b - tartrate-resistant acid phosphatase 5b

As a further step to evaluate independent predictors of post-intervention bone marker levels, a general linear model was conducted including treatment group, sex, and GFR stage as predictors while controlling for baseline values.

After adjustment for baseline values, using a general linear model, the overall adjusted mean intact PINP was 78.7 µg/L (95% CI 67.1–90.3). No significant differences were observed between treatment groups (*p* = 0.50), sexes (*p* = 0.12), or GFR stages (*p* = 0.54). Baseline PINP showed a trend toward predicting post-intervention levels (*p* = 0.056). For TRAP5b, the adjusted mean was 1.87 U/L (95% CI 1.61–2.13), with no significant effects of treatment group (*p* = 0.29), sex (*p* = 0.24), or GFR stage (*p* = 0.85).

### Intact PINP and TRAP5b levels compared to healthy pre-menopausal reference values

Using the medians of healthy pre-menopausal women as cutoffs, intact PINP levels above 36 µg/L were observed in most of patients at the start and end of the study (87.6% and 88.4%, respectively). However, most patients had TRAP5b levels below 3.1 U/L at both the start and end of the study period (71.9% and 93.6%, respectively).

### Laboratory correlates of bone marker changes

There were no significant between-group differences for Δ intact PINP or Δ TRAP5b. Δ intact PINP showed a positive trend in the strength group and a negative trend in the balance group (12.8 [-89–112] vs. -5.9 [-349–152], *p* = 0.07). Δ TRAP5b exhibited a slight negative trend in both groups (-0.1 [-5.5–2.6] vs. -0.3 [-6.6–3.7], *p* = 0.82).

Significantly higher levels of phosphate and parathyroid hormone after 12 months were observed in patients with positive Δ intact PINP as well as positive Δ TRAP5b. Higher 25(OH)D was observed in participants with negative Δ TRAP5b values (Table 4).

**Table 4:** Changes in some laboratory analyses according to changes in delta values of measured markers of bone turnover

	T0	T12	*P*
**Δ intact PINP decreased** (***n*** **= 46**)			
P-calcium (mmol/L)	2.3 ± 0.1	2.3 ± 0.0.1	0.44
P-phosphate (mmol/L)	1.2 ± 0.2	1.2 ± 0.2	0.47
P-PTH (pmol/L)	11 [3–135]	13.5 [1.7–59]	0.25
P-ALP (µkat/L)	1.4 ± 0.5	1.3 ± 0.3	0.17
P-25(OH)D (nmol/L)	59.2 ± 25	66.8 ± 30	0.14
**Δ intact PINP increased** (***n*** **= 45**)			
P-calcium (mmol/L)	2.3 ± 0.1	2.3 ± 0.1	0.21
P-phosphate (mmol/L)	1.1 ± 0.2	1.2 ± 0.3	**0.01**
P-PTH (pmol/L)	11 [2.6–26]	11 [1.8–36]	**0.03**
P-ALP (µkat/L)	1.3 ± 0.5	2.4 ± 6.6	0.30
P-25(OH)D (nmol/L)	68.4 ± 26	71.9 ± 28	0.35
**Δ TRAP5b decreased** (***n*** **= 53**)			
P-calcium (mmol/L)	2.3 ± 0.1	2.3 ± 0.1	0.99
P-phosphate (mmol/L)	1.2 ± 0.2	1.1 ± 0.23	0.62
P-PTH (pmol/L)	11 [4–133]	12 [1.7–48]	0.30
P-ALP (µkat/L)	1.3 ± 0.4	2.2 ± 6.1	0.33
P-25(OH)D (nmol/L)	62.0 ± 28	69.3 ± 27.0	**0.03**
**Δ TRAP5b increased** (***n*** **= 38**)			
P-calcium (mmol/L)	2.3 ± 0.1	2.3 ± 0.1	0.70
P-phosphate (mmol/L)	1.1 ± 0.2	1.2 ± 0.3	**0.02**
P-PTH (pmol/L)	11 [2.6–45]	12.5 [3.5–59]	**0.01**
P-ALP (µkat/L)	1.4 ± 0.5	1.4 ± 0.4	0.99
P-25(OH)D (nmol/L)	64.8 ± 21.9	68.2 ± 31.1	0.55

Data presented as mean ± SD or median with minimal - maximal values. P- plasma, PTH– parathyroid hormone, ALP- alkaline phosphatase, 25(OH)D- 25 -hydroxy-vitamin D, Intact PINP- N-terminal propeptide of type I procollagen, intact, TRAP5b - tartrate-resistant acid phosphatase 5b

Most patients had calcium values within reference intervals in both the increased and decreased intact PINP groups (95.7% vs. 90.9%, *p* = 0.10), with no hypercalcemia observed. Hyperphosphatemia was rare (10.5% vs. 7%, *p* = 0.72), and ALP values were predominantly normal (88.9% vs. 90.9%, *p* = 1.0). Only a minority had 25(OH)D levels above 75 nmol/L (37.2% vs. 28.6%, *p* = 0.62). Elevated PTH (> 6.9 pmol/L) was common (88.6% vs. 78.3%), and no patients had PTH below 1.6 pmol/L.

In the TRAP5b groups, all patients in the increased group and most in the decreased group had calcium values within reference intervals (100% vs. 90.4%, *p* = 0.15), with two patients (3.8%) in the decreased TRAP5b group experiencing hypercalcemia. Hyperphosphatemia was rare (11.1% vs. 7.7%, *p* = 0.71), and ALP values were mostly normal (89.2% vs. 90.4%, *p* = 0.71). Only a minority had 25(OH)D > 75 nmol/L (29.7% vs. 34%, *p* = 0.15), and elevated PTH was common in both groups (83.3% vs. 82.7%, *p* = 1.00), with no patients below the lower reference limit.

No differences were observed in prescription of active vitamin D in patients with positive vs. negative Δ intact PINP (59% vs. 65% accordingly, *p* = 0.66). There were no statistically significant differences in frequency of prescription of active vitamin D in patients with positive vs. negative Δ TRAP5b values in either group (65% vs. 60%, *p* = 0.66).

### Bone turnover categories

**Fig. 4:**
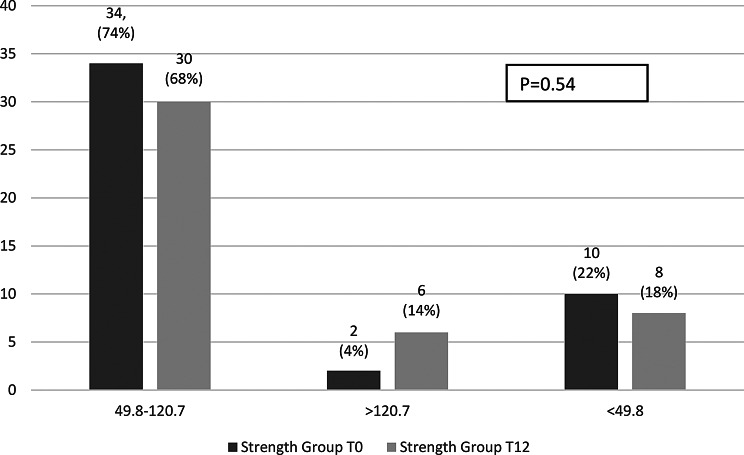
Proportion of participants across intact PINP categories at baseline and 12 months in strength group

**Fig. 5:**
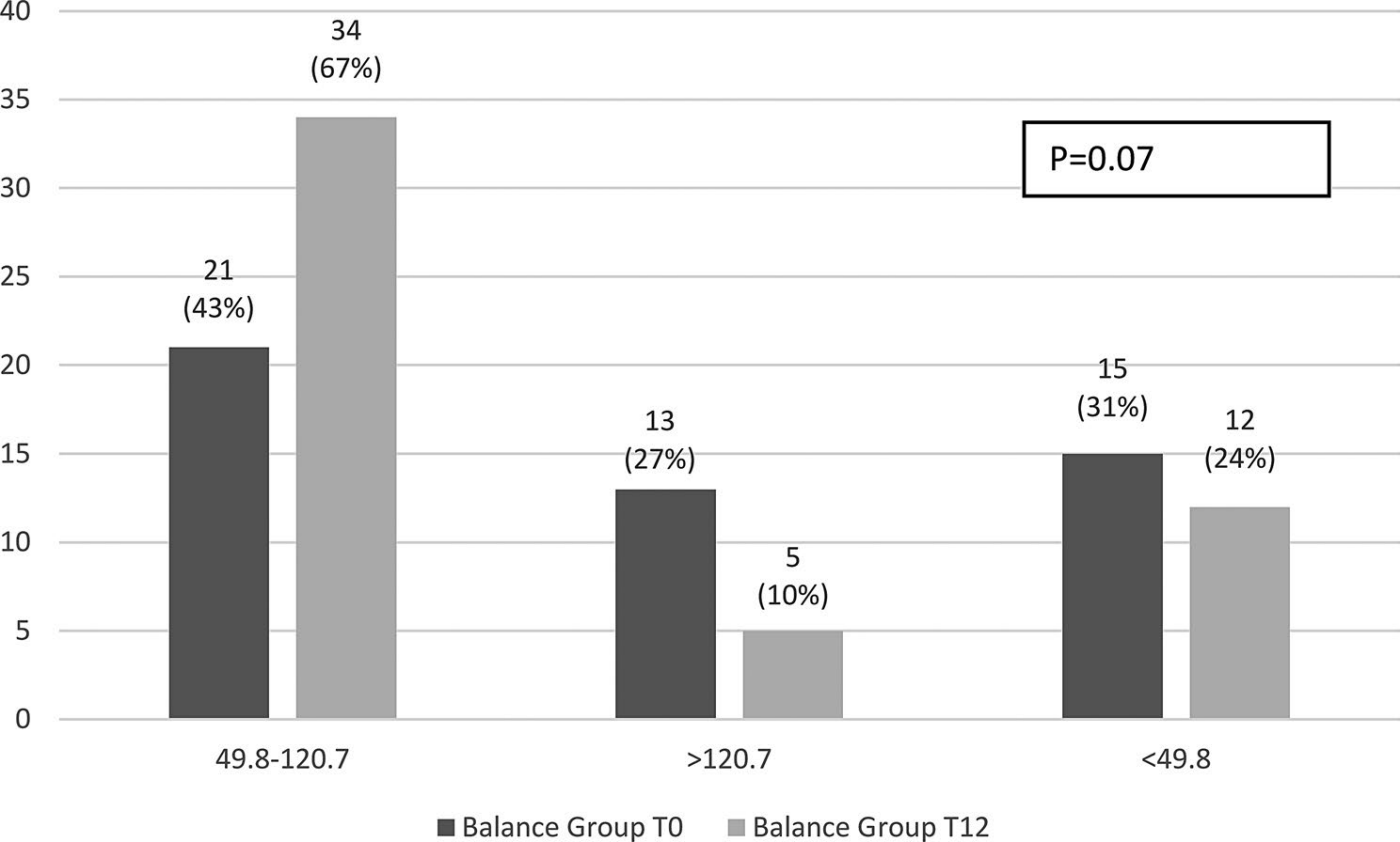
Proportion of participants across intact PINP categories at baseline and 12 months in the balance group

The proportion of participants with intact PINP levels indicating high bone turnover (> 120.7 µg/L) showed a tendency to increase in the strength group (4% at baseline to 14% at 12 months, *p* = 0.54) and decrease in the balance group (27% to 10%, respectively, *p* = 0.07), without reaching statistical significance (Figs. 4 and 5).

**Fig. 6:**
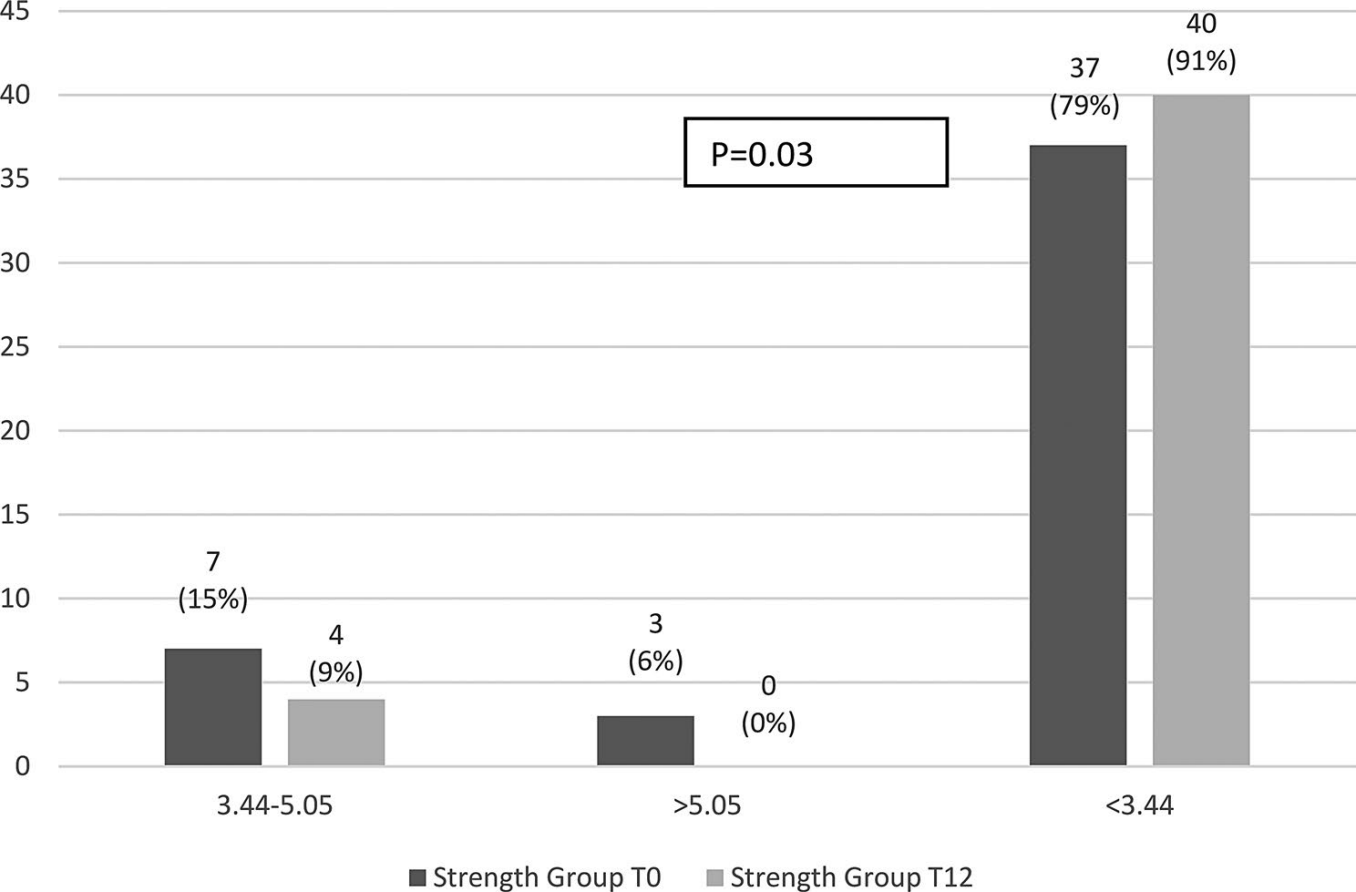
Proportion of participants across TRAP5b categories at baseline and 12 months in strength group

**Fig. 7:**
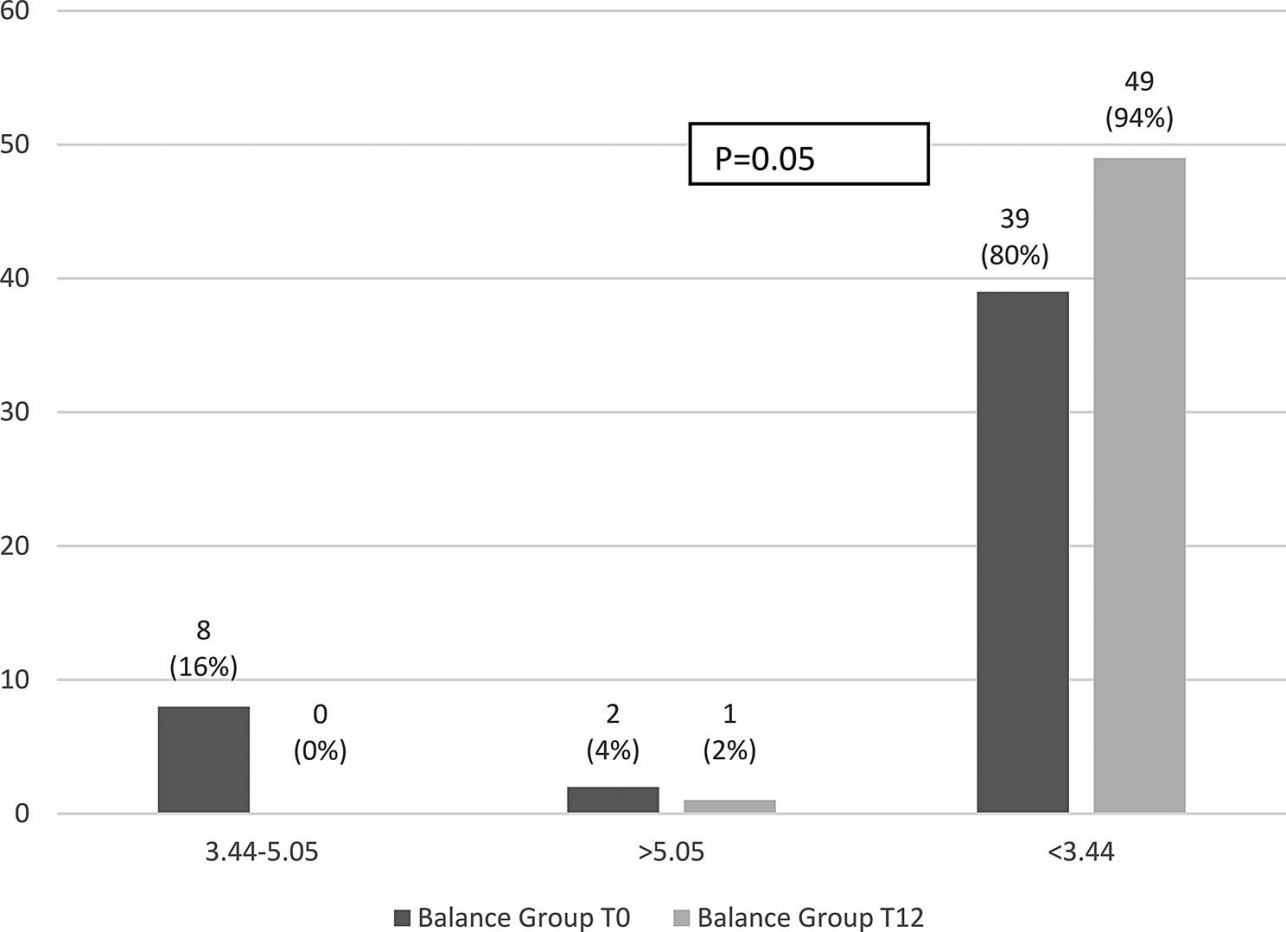
Proportion of participants across TRAP5b categories at baseline and 12 months in the balance group

In contrast, the proportion with TRAP5b levels consistent with low bone turnover (< 3.44 U/L) rose significantly in both groups: from 79% at baseline to 91%at 12 months in the strength group (*p* = 0.03) and from 80% to 94%, respectively in the balance group (*p* = 0.05) (Figs. 6 and 7).

## Discussion

We investigated the impact of exercise on markers of bone formation and resorption, attempting to increase understanding of its role in bone metabolism. In this cohort of patients with advanced CKD, 12 months of strength or balance exercise improved physical performance but had minimal impact on bone turnover markers. However, both interventions were associated with more participants exhibiting TRAP5b values consistent with low bone turnover, indicating a potential role in mitigating bone resorption.

Few studies have assessed bone-specific markers in response to exercise in CKD patients. Most previous research focused on patients on hemodialysis and involved small sample sizes [6, 7]. These studies generally reported improvements in bone mineral density without significant changes in bone turnover markers, highlighting the uncertainty of exercise effects on bone metabolism in CKD. By contrast, studies in non-CKD populations suggest that exercise can stimulate bone formation and transiently reduce bone resorption. For instance, resistance exercise in healthy young men increased markers of bone formation while suppressing resorption [9], and lifestyle interventions in individuals with type 2 diabetes led to balanced increases in both formation and resorption markers, including PINP, osteocalcin, and CTX-I [20]. Similarly, walking interventions in osteopenic women increased PINP, CTX, and osteocalcin [21]. These observations support the idea that mechanical loading can influence bone turnover, although CKD-related alterations may blunt this response.

A notable finding in our study was the significant increase in the proportion of TRAP5b values consistent with low bone turnover (TRAP5b < 3.44 U/L) in both the strength and balance groups. This increase suggests that both interventions might have effectively contributed to reducing bone resorption. Our results are in line with the findings of studies in healthy men or patients with osteoporosis linking exercise to increased markers of bone formation, while transiently suppressing a marker of bone resorption [9, 22]. Low bone turnover is typical in the early stages of CKD and slowly proceeds to high turnover in the later stages of CKD. Thus, our findings showing an increase in TRAP5b values, which are consistent with low turnover, could be considered a sign of a protective effect of exercise from a bone perspective.

Although the frequency of hyperphosphatemia and elevated PTH did not differ between participants with increased versus decreased Δ intact PINP and Δ TRAP5b, higher average phosphate and PTH levels were observed in the groups with rising bone turnover markers. Participants with decreases in TRAP5b had higher 25(OH)D levels, suggesting a potential protective role of vitamin D in bone resorption. Findings from the literature on this subject are not uniform. Direct effects of PTH on osteoblasts and osteocytes and indirect actions on osteoclasts promote both bone formation and bone resorption. Bone resorption predominates in response to continuous exposure to high levels of circulating PTH [23]. A previous study has reported that higher phosphate levels may induce osteoblast apoptosis and reduce bone formation as well as inhibit bone resorption [24]. One could speculate that this direct effect of high phosphate could be ameliorated by an indirect stimulating effect on PTH secretion as well as suppressing 1-alpha-hydroxylase activity contributing to calcitriol deficiency [24]. On the other hand, a recent study in patients on hemodialysis revealed that high serum phosphate was consistently associated with an increased risk of bone fractures, regardless of other factors such as previous fractures, age, sex, time on hemodialysis, serum calcium and PTH [25].

According to our data, increasing 25(OH)D levels could be associated with a decrease in bone resorptive activity. It is notable that despite a significant increase in 25(OH)D levels after 12 months the recommended levels of 75nmol/L were not reached in most patients. Direct effects of vitamin D on bone include activities on both formation and resorption. Conflicting data suggest that these actions may differ by skeletal site and dietary calcium intake [26]. In the case of a sufficient calcium supply, vitamin D and its metabolites can improve calcium balance and facilitate mineral deposition in bone matrix largely without direct effects on bone cells, although some beneficial effects may occur via mature osteoblasts, as demonstrated in mice with osteoblast-specific overexpression of VDR or 1α-hydroxylase [26]. In the setting of calcium deficiency, however, calcitriol enhances bone resorption, whereas simultaneously inhibiting bone mineralization, to maintain serum calcium homeostasis at the expense of bone mass [26].

The levels of both markers in our study are significantly lower compared with the levels presented by Jørgensen et al. [2]. Salam et al. reported higher TRAP5b levels but similar levels of intact PINP levels as in our study [27]. These differences can at least partly be explained by differences in study population. In Jørgensen’s et al. study 18% of the patients were in CKD stages 4–5, the rest were on KRT [2]. In Salam’s et al. study 63% of the patients were in stages 4–5 of CKD and 27% were on KRT [27]. Salam and al have also showed that patients with CKD had significantly higher marker levels compared with controls which could not only be explained by accumulation due to decreased kidney function as both the markers tested are known not to be influenced by decreased excretion. One could speculate that there are different patterns of bone turnover as CKD progresses.

In our CKD G3–5 patients not on dialysis, most had TRAP5b levels below and intact PINP levels above the medians of healthy pre-menopausal women, suggesting low bone resorption alongside relatively high bone formation compared with a healthy population. This pattern may support the concept that bone turnover changes dynamically as CKD progresses.

Evidence suggests that in the early stages of CKD, low bone turnover is often the earliest detectable alteration in bone remodeling [28]. This may be due to a predominance of factors that inhibit bone turnover, such as resistance to PTH, low calcitriol levels, sex hormone deficiency, diabetes, and uremic toxins. These factors contribute to the suppression of osteocyte Wnt/b-catenin signaling and the increased expression of Wnt antagonists like sclerostin, Dickkopf-1, and sFRP4. High turnover bone disease typically emerges at later stages of CKD, when serum PTH levels can overcome peripheral PTH resistance and other inhibitors of bone formation [28].

There are several strengths in this study. The participants represent a typical cohort of patients with CKD stages 3 to 5 not on KRT and, to our knowledge, this is the first study to compare effects of strength versus balance exercise on bone specific markers in patients with CKD not on KRT. This study also has limitations such as the absence of a sedentary control group, but the reasons for that have been discussed and explained previously [10]. Additionally, the short follow-up period could be considered a significant limitation as the intervention that lasted 12 months is acceptable for investigating effects of exercise in patients with CKD but might be insufficient to capture meaningful changes in bone metabolism in this group. Bone remodeling is a slow process, and longer observation periods are likely necessary to detect significant shifts in bone metabolism reflecting markers.

Moreover, we were unable to examine bALP, a key marker of bone formation and skeletal mineralization which limits the comprehensiveness of the bone status assessment. This limitation at least partly was compensated by analysis of total ALP which did not reveal any significant differences.

In conclusion 12 months of strength or balance exercise improved physical performance but had minimal impact on bone turnover markers. However, both exercise modalities were associated with a trend toward reduced bone resorption, as indicated by an increased proportion of patients with TRAP5b values consistent with low turnover.

These findings warrant further investigation into the long-term effects of exercise on bone health in CKD, with a particular focus on the mechanisms underlying these responses.

## Data Availability

The datasets used and/or analyzed during the current study are available from the corresponding author on reasonable request. All data are stored on a server managed by the Southern Health Care Region.

## Abbreviations

CKD: Chronic Kidney Disease
PTH: Parathyroid Hormone
Balp: Bone Specific Alkaline Phosphatase
KDIGO: Kidney Disease-Improving Global Outcomes
PINP: Procollagen Type I N-terminal Propeptide
TRAP5b: Tartrate-Resistant Acid Phosphatase Isoform 5b
KRT: Kidney Replacement Therapy
RENEXC: RENal EXerCise
RPE: Borg Rating of Perceived Exertion
6-MWT: 6-Minute Walk Test
30-STS: 30-Seconds Sit-to-Stand Test
FR: Functional Reach
ISQ: Isometric Quadriceps Strength
GFR: Glomerular Filtration Rate
ALP: Alkaline Phosphatase
SWEDAC: Swedish Board for Accreditation and Conformity Assessment
25(OH)D: Plasma 25-Hydroxyvitamin D
SPSS: Statistical Package for the Social Sciences
CONSORT: Consolidated Standards of Reporting Trials
ISO: International Organization for Standardization
